# Potent Inhibition of Junín Virus Infection by Interferon in Murine Cells

**DOI:** 10.1371/journal.pntd.0002933

**Published:** 2014-06-05

**Authors:** Cheng Huang, Aida G. Walker, Ashley M. Grant, Olga A. Kolokoltsova, Nadezhda E. Yun, Alexey V. Seregin, Slobodan Paessler

**Affiliations:** Department of Pathology and Institute for Human Infections and Immunity, University of Texas Medical Branch, Galveston, Texas, United States of America; George Mason University, United States of America

## Abstract

The new world arenavirus Junín virus (JUNV) is the causative agent of Argentine hemorrhagic fever, a lethal human infectious disease. Adult laboratory mice are generally resistant to peripheral infection by JUNV. The mechanism underlying the mouse resistance to JUNV infection is largely unknown. We have reported that interferon receptor knockout mice succumb to JUNV infection, indicating the critical role of interferon in restricting JUNV infection in mice. Here we report that the pathogenic and vaccine strains of JUNV were highly sensitive to interferon in murine primary cells. Treatment with low concentrations of interferon abrogated viral NP protein expression in murine cells. The replication of both JUNVs was enhanced in IRF3/IRF7 deficient cells. In addition, the vaccine strain of JUNV displayed impaired growth in primary murine cells. Our data suggested a direct and potent role of host interferon response in restricting JUNV replication in mice. The defect in viral growth for vaccine JUNV might also partially explain its attenuation in mice.

## Author Summary

The new world arenavirus Junín virus (JUNV) is the causative agent of a lethal human infectious disease, Argentine hemorrhagic fever. Laboratory mice are used as models to study many viral diseases. However, adult laboratory mice are generally resistant to JUNV infection. Interferons are early immune regulatory molecules that induce potent anti-viral status in host cells and activate host immune cells to counteract virus infection. The activity of interferons relies on their cell surface receptors. We have previously reported that mutant mice with defect in interferon receptors succumbed to challenge with JUNV, highlighting the critical role of interferon in restricting JUNV infection in mice. Here we further study the basis of mouse resistance to JUNV infection and report that the replication of both pathogenic JUNV and its vaccine strains are highly sensitive to type I IFN treatment in mouse cells. However, both strains replicate efficiently in Africa green monkey-derived Vero cells and human cells when treated with high doses of interferon. Additionally, the vaccine strain replicates less efficiently in mouse cells compared with the pathogenic strain, which might partially explain its attenuation in mice. Our new findings help better understand the JUNV-host interaction.

## Introduction

Arenaviruses are enveloped RNA viruses with bi-segmented, negative-sense genomic RNA [1]. Based on the antigenicity, phylogeny, and geographical distribution, they are divided into the Old World (Lassa-Lymphocytic choriomeningitis complex) arenaviruses and the New World (Tacaribe complex) arenaviruses. Recent studies have identified several snake-borne arenaviruses that are highly divergent from known arenaviruses [2]–[4]. The lymphocytic choriomeningitis virus (LCMV) from the Old World (OW) arenaviruses is the prototype arenavirus. The New World (NW) arenaviruses are further classified into clades A, B, and C NW arenaviruses. Arenaviruses often chronically infect their natural rodent hosts [1]. Infection in humans is mostly acute and occurs probably through mucosal exposure to aerosols or by direct contact of abraded skin with infectious materials.

The *Arenaviridae* family includes several important human pathogens [1], [5], [6]. The OW Lassa virus (LASV) is the causative agent of Lassa fever, a major public health concern in western Africa [7]. Several clade B NW arenaviruses, including Junín virus (JUNV), Machupo virus (MACV), Guanarito virus (GTOV), Sabia virus (SABV) and Chapare virus (CHAV), cause human hemorrhagic fever diseases in South America [1], [5]–[8]. JUNV is the causative agent of Argentine hemorrhagic fever [1], a highly infectious human disease with 15–30% case fatality [8]–[12], meanwhile JUNV could induce lethal, transient or persistent infection in its natural rodent host, *Calomys musculinus* [1], [13]. Field and laboratory studies have demonstrated that JUNV infection in its primary natural rodent host is largely through horizontal transmission by close contact and that rodents undergoing persistent infection could shed a large amount of virus in saliva and urine [12], [14]. JUNV is classified as a select agent by the Centers for Diseases Control and Prevention in the United States. Research work utilizing infectious JUNV requires a high-containment biosafety level 4 facility in the USA. A live attenuated vaccine Candid #1 was developed as a collaborative effort by the US Army Medical Research Institute of Infectious Diseases (USAMRIID) and the Argentine Ministry of Health and Social Action [15], [16]. The original human pathogenic XJ strain had been serially passaged in guinea pigs and mouse brains. The resulting XJ44 strain was attenuated for guinea pigs and humans but not for young mice infected intracranially. After additional serial passages in FRhL-2 cells, the virus eventually became attenuated in young mice and was selected as the final Candid #1 vaccine stock.

Adult laboratory mice (older than 21-days) are generally resistant to peripheral infection by any JUNV [6], [12], [13]. Suckling mice are vulnerable to lethal virus challenge intracranially and mainly develop neurotropic and immunopathological diseases distinct from symptoms observed in patients and in experimentally infected nonhuman primates and guinea pigs [12]. The mechanism underlying the mouse resistance to JUNV infection is largely unknown. Previously, we have reported that mice lacking type I and type II interferon (IFN) receptors succumbed to lethal JUNV infection, which provides a novel model that recapitulates some symptoms found in AHF patients [17]. This result demonstrated the critical role of IFN pathway in restricting JUNV infection in mice and prompted us to further explore whether IFN could directly inhibit JUNV replication in murine cells. In this study, we provide evidence showing that both pathogenic and vaccine strains of JUNV were highly sensitive to interferon treatment in murine primary cells. The multiplication of JUNVs was enhanced in IRF3/IRF7 deficient cells. In addition, the vaccine strain JUNV displayed impaired growth in murine primary cells, which could partially explain the attenuation of virus.

## Methods

### Viruses and cells

The pathogenic strain Romero JUNV was obtained from Dr. Thomas G. Ksiazek (Centers for Disease Control and Prevention, Atlanta, GA). The vaccine strain Candid#1 JUNV and Vesicular stomatitis virus were provided by Dr. Robert Tesh (The World Reference Center for Emerging Viruses and Arboviruses (WRCEVA), University of Texas Medical Branch, Galveston, TX). Virus stocks were propagated on Vero cells (American Tissue Culture Collection, Manassas, VA), followed by filtration through filters (0.45 µm pore size) to remove cell debris and by purification with Ultra 100 K Filters Devices (Ultralcel 100 K, molecular weight cutoff 100,000, Amicon, Millipore). Human lung epithelial A549 cells were obtained from ATCC. Primary mouse embryonic fibroblast cells derived from wild type C57BL/6 mice and IRF3/7 knockout mice were provided by Dr. Michael Diamond (Washington University). All work with the pathogenic Romero strain JUNV was performed in the University of Texas Medical Branch BSL-4 facilities (the Galveston National Laboratory) in accordance with institutional health and safety guidelines and federal regulations as described previously [18].

### Virus sensitivity to IFN treatment

Vero and A549 cells were seeded into 96-well plates for 24 h and treated with human IFN-α-2b (Intron A, Schering Corporation, NJ), IFN-β1a (PBL, NJ) or IFN-γ (Sigma-Aldrich, MO) at 125, 250, 500 and 1000 U/ml for 16 h. MEF cells were treated with mouse IFN-β (PBL) as indicated. Cells were then infected with VSV, Candid#1 JUNV or Romero JUNV at an MOI of 0.1 PFU/cell. IFNs were supplemented after virus infection. For Romero and Candid#1 JUNV infection, supernatants were collected at 3 days post infection and assayed for virus production by plaque assay. For VSV infection, supernatants were collected at 16 hr p.i.. Data represent the mean of three experiments ±SEM.

### Western blotting

Cells were seeded into 12-well plates for 24 h and then treated with various concentrations of human IFN-β1a or mouse IFN-β (PBL) as indicated in each experiment. Cells were infected with Candid#1 JUNV at an MOI of 3 PFU/cell. IFNs were supplemented after virus infection. Protein lysates were prepared in 2x Laemmli sample buffer at 1 and 2 days p.i. from MEF cells and A549 cells, or from Vero cells at 2 days p.i.. Protein samples were resolved on 4–20% SDS-PAGE gel and transferred to PVDF membranes using Mini Trans-Blot Electrophoretic Transfer Cell apparatus (Bio-Rad, CA). Membranes were incubated with primary antibodies overnight at 4°C and then with appropriate secondary antibodies for 1 h at room temperature. Proteins were visualized with ECL Western Blotting Detection Reagents (GE, NJ) according to the manufacturer's instruction. Viral NP protein was detected with a monoclonal mouse anti-JUNV NP antibody (AG12, BEI). Equal loading of samples was confirmed by immunoblotting of the same membranes with an antibody to β-actin (sc-1616, Santa Cruz). Secondary antibodies HRP-conjugated Goat anti-mouse IgG (115-035-146, Jackson Immunology) and HRP-conjugated donkey anti-goat IgG (sc-2020, Santa Cruz) were used.

### Growth kinetics of JUNV in wild-type MEF and IRF3/7 knockout MEF

Wild-type MEF cells and IRF3/7 knockout MEF cells were infected by Romero and Candid#1 viruses at MOI of 0.1 or 0.001. Supernatants from infected cells were harvested daily and subjected to plaque assay as described previously [18]. Statistical analysis of virus growth kinetics was performed by two way ANOVA test.

## Results

### Effect of interferon treatment on JUNV multiplication in murine cells

**Figure 1:**
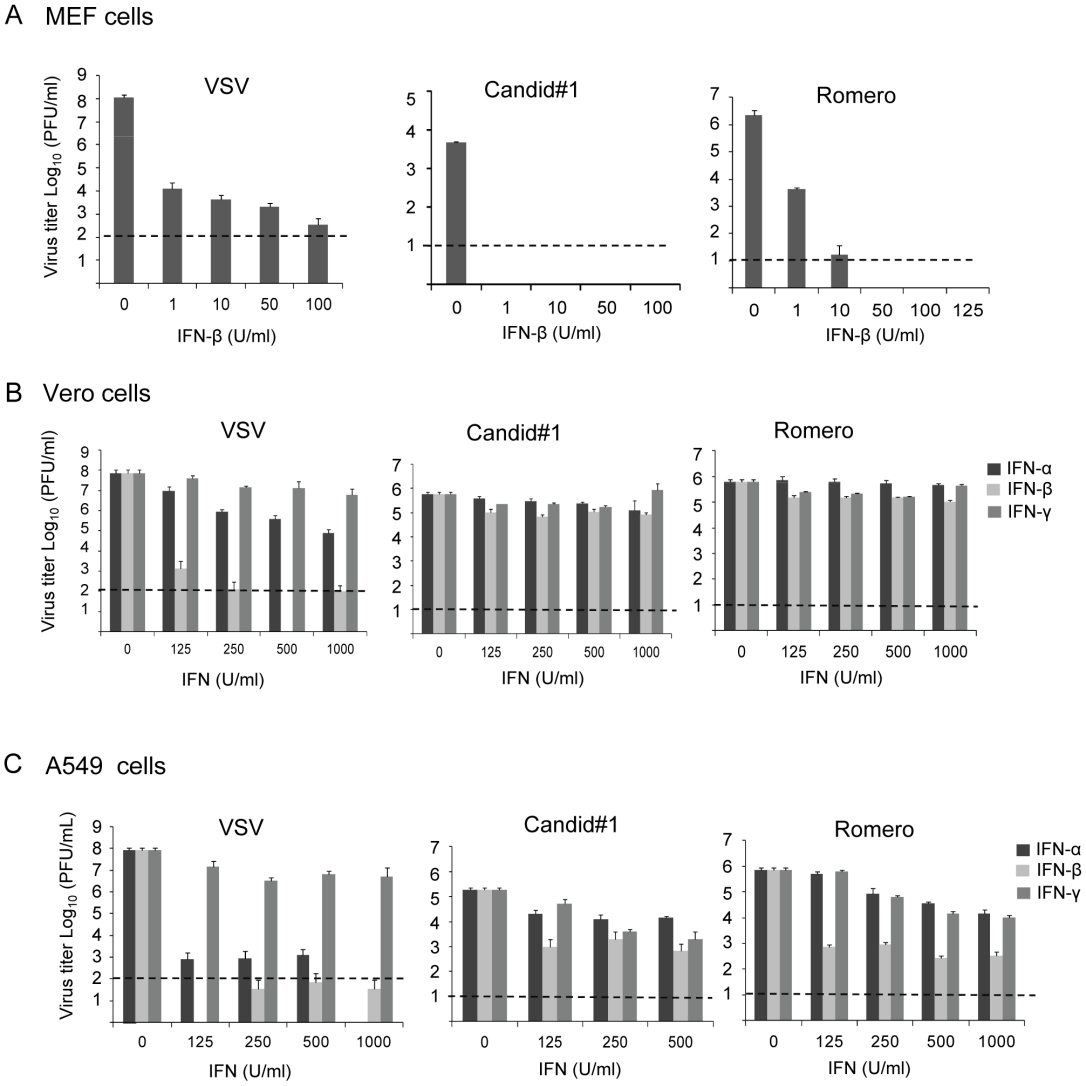
Interferon sensitivity of JUNV in different cell lines. (A) mouse embryonic fibroblast cells (MEF), (B) Vero cells (ATCC) and (C) human lung carcinoma epithelial A549 cells (ATCC) were treated with IFNs at the indicated concentrations for 16 h. Vero cells and A549 cells were treated with human IFN-α2b (Schering), IFN-β (PBL) or IFN-γ (Sigma), while MEF cells were treated with mouse IFN-β (PBL), respectively. Cells were then infected with VSV, Candid#1 JUNV or Romero JUNV at an MOI of 0.1 PFU/cell. IFNs were supplemented after virus infection. During Romero and Candid#1 JUNV infection, supernatants were collected at 3 days p.i. and assayed for virus production by plaque assay. During VSV infection, supernatants were collected at 16 h.p.i.. Dotted lines indicate the limitation of plaque assay. Data represent the mean of three experiments ±SEM.

To understand if IFN has direct impact on JUNV infection, we characterized the effects of IFN treatment on JUNV multiplication in primary murine embryonic fibroblast cells (MEF) derived from C57BL/6 mice, Vero cells or human lung epithelial A549 cells. MEF cells were treated with mouse IFN-β at 1, 10, 50 or 100 U/ml for 16 hrs before and after virus infection, meanwhile Vero cells and A549 cells were treated with human IFN-α, IFN-β or IFN-γ for 16 hrs before and after infection at 125, 250, 500 or 1000 U/ml (Fig 1). Cells were then infected with the pathogenic Romero strain or the vaccine strain Candid#1 JUNV at a multiplicity of infection (MOI) of 0.1 PFU/cell. At 72 hr post infection (p. i.) virus titers in tissue culture supernatants were determined by plaque assay. We also included the IFN-sensitive VSV as a control. The result showed that IFN-β mediated a potent antiviral effect against both Romero and Candid#1 JUNV infection in MEF cells (Fig 1A). Notably a low dose of murine IFN-β treatment (1 U/ml) resulted in drastic decrease in virus titers by over 3-log for Candid#1 and by 2.7-log for Romero JUNV. Meanwhile, the titer of VSV was reduced by 4-log at 1 U/ml (Fig 1A). The multiplication of Candid#1 was completely abolished in MEF cells treated with 1 U/ml IFN-β, while the multiplication of Romero virus was abolished at 50 U/ml (Fig 1A), demonstrating the high sensitivity of JUNV to IFN-mediated antiviral effect in murine cells. In comparison, the virus titers of some IFN-sensitive LCMV strains are decreased by approximately 2-log when treated with 100 U/ml murine IFN-α/β in murine cells [19].

In IFN-α/β gene defective Vero cells, the titers of JUNV were reduced by less than 1-log when treated with a high concentration of human IFN-α, β or γ (1000 U/ml) (Fig 1B), which was consistent with our previous studies [20]. In comparison, the titer of VSV was remarkably reduced by close to 5-log in the presence of 125 U/ml human IFN-β, indicating the relative insensitivity of both JUNV strains to IFN in Vero cells. In human A549 cells, treatment with 500 U/ml human IFN-β suppressed virus growth by 2-log and 3-log for Candid#1 strain and Romero strain, respectively, while treatment with 125 U/ml human IFN-β reduced virus titer by more than 6-log for VSV (Fig 1C). This result showed that both strains of JUNV were relatively more sensitive to IFN in A549 cells than in Vero cells, but less susceptible to IFN than the IFN-sensitive VSV.

### JUNV growth in wt MEF and IRF3/IRF7 knockout MEF

**Figure 2:**
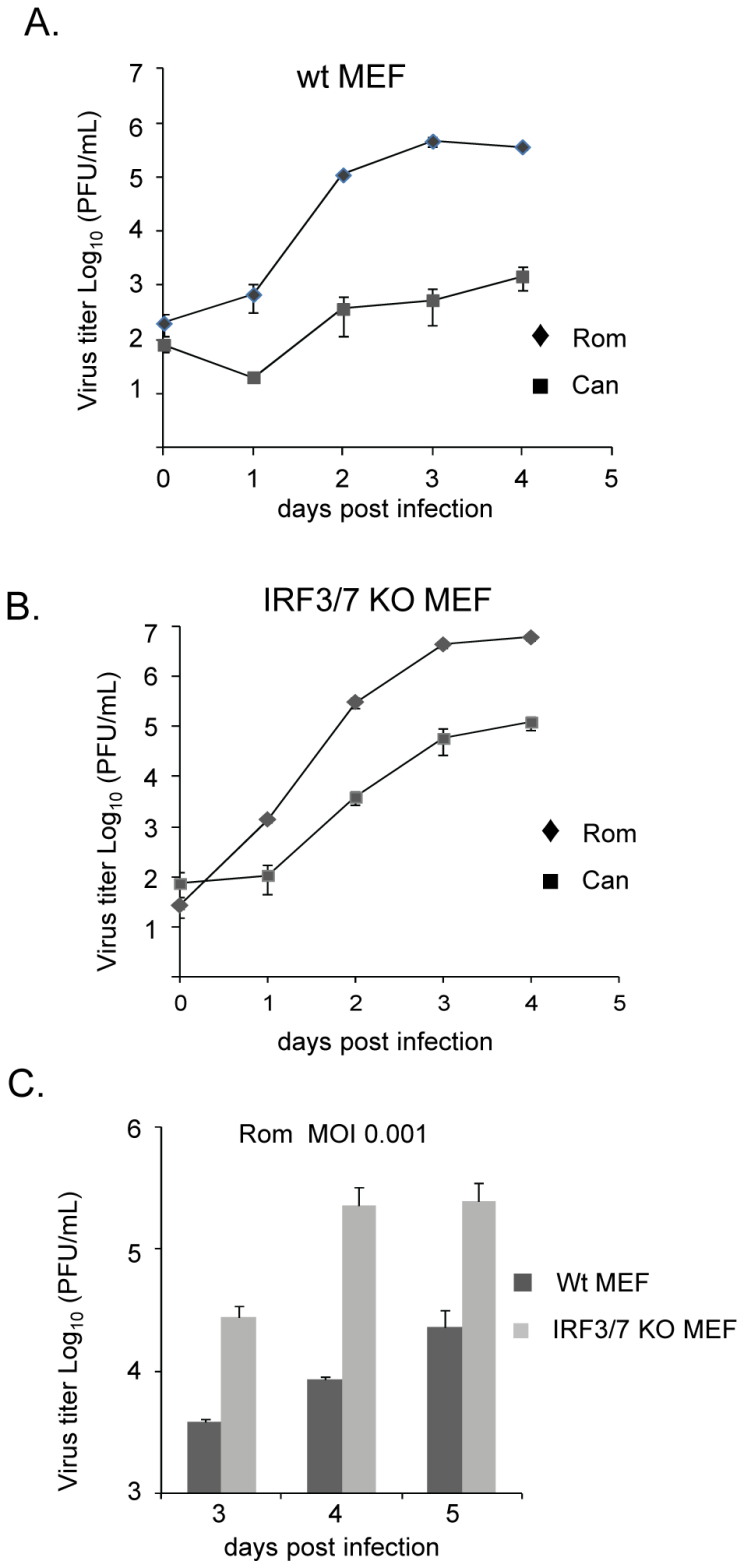
Growth kinetics of JUNV in wild-type MEF and IRF3/7 knockout MEF. Wild-type MEF cells (A) and IRF3/7 knockout MEF cells (B) were infected by Romero (Rom) and Candid#1 (Can) viruses at an MOI of 0.1. (C) Wild-type MEF cells and IRF3/7 knockout MEF cells were infected by Romero virus at an MOI of 0.001. Supernatants from infected cells were harvested daily and subjected to plaque assay. Note: the titer of Candid#1 virus was below the detection level in both MEFs at an MOI of 0.001. Data represent the mean of triplicates ±SEM. Wt MEF: Wild-type MEF cells. IRF3/7 KO MEF: IRF3/7 knockout MEF cells.

It seems that the pathogenic Romero virus replicated more efficiently than the vaccine strain Candid#1 virus did in wild-type MEF cells (Fig 1A). We characterized JUNV growth in primary MEF cells at an MOI of 0.1 and found that the Romero virus indeed multiplied significantly greater than the Candid#1 virus (Fig 2A, *P*<0.001, two way ANOVA test). The peak titer of Romero virus (3.6×10^5^ PFU/ml) was about 240-fold of that of Candid#1 virus (1.5×10^3^ PFU/ml) at 4 d.p.i. (Fig 2A). JUNV growth was further examined in IRF3/IRF7 double knockout (KO) MEF cells (MOI = 0.1) where the IFN response is largely abrogated [21]. While both strains showed enhanced growth in IRF3/7 KO MEF cells (Fig 2B), the Romero virus replicated more efficiently than the vaccine Candid#1 virus (*P*<0.001, two way ANOVA test). The peak titer of Romero virus (6.2×10^6^ PFU/ml) was 47-fold of that of Candid#1 virus (1.3×10^5^ PFU/ml) at 4 d.p.i.. The difference was largely comparable to that in wild-type MEF (Fig 2A), implying the impaired growth of Candid#1 virus was less likely associated with host IFN response but more likely due to its intrinsic growth deficiency in primary MEF cells. We further examined JUNV growth in MEF cells at lower MOI (MOI 0.001), a condition more mimicking virus infection *in vivo* (Fig 2C). As expected, Romero virus grew at lower peak titers in both cell lines than it did at MOI of 0.1. We again observed more productive multiplication for Romero virus in IRF3/7 KO MEF cells (2.5×10^5^ PFU/ml, 5 d.p.i.) than in wt MEF (2.3×10^4^ PFU/ml, 5 d.p.i.), supporting the role of IFN pathway in suppressing JUNV infection in murine cells. However, the growth of Candid#1 virus was below the detection level in wt MEF or IRF3/7 KO MEF cells at MOI of 0.001, demonstrating the impaired Candid#1 virus growth in primary murine cells as compared with the Romero virus regardless of the integrity of the IFN pathway.

### Effect of IFN treatment on JUNV replication

**Figure 3:**
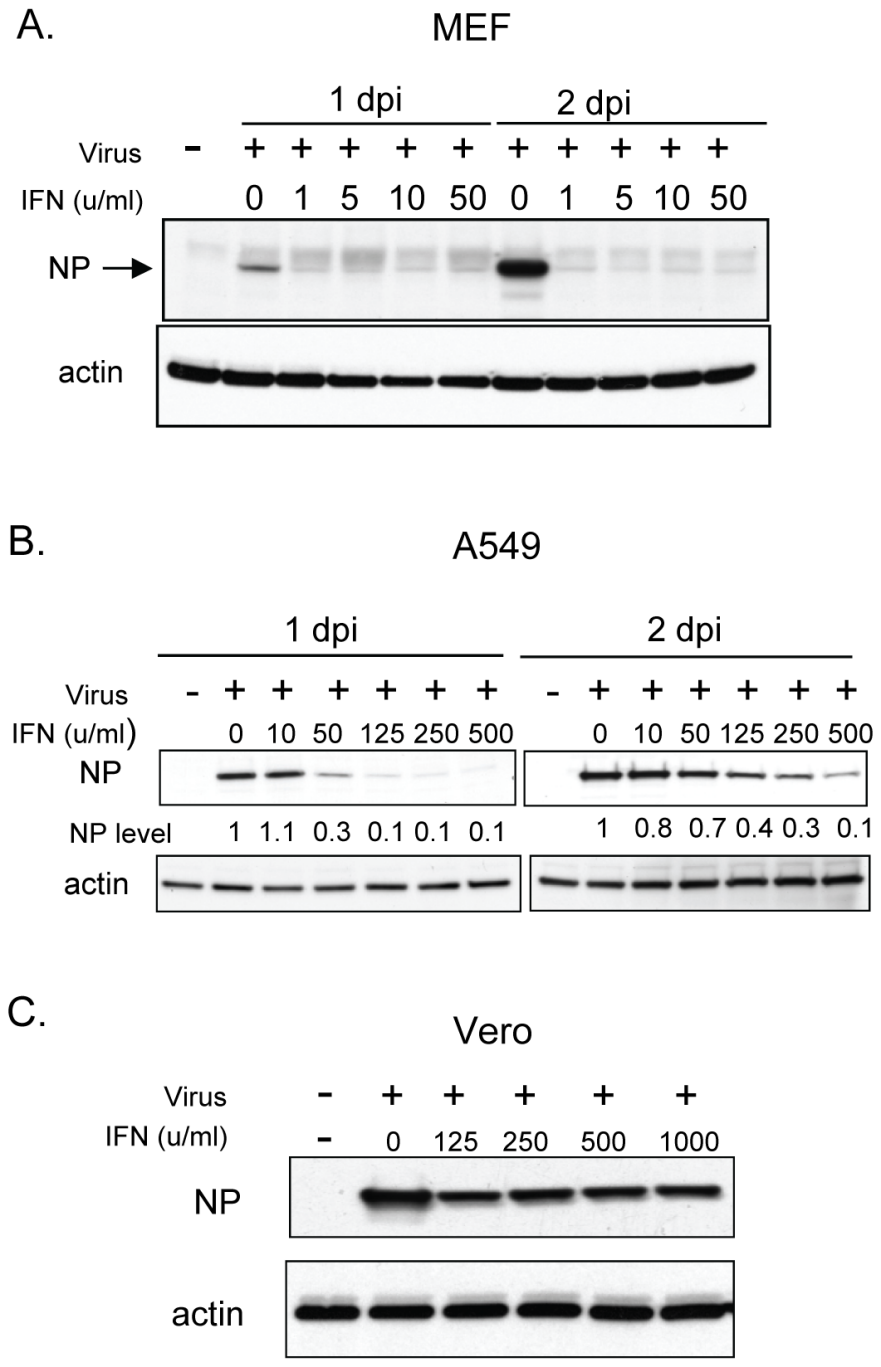
Immunoblotting analysis of viral protein expression. MEF cells (A), A549 cells (B) and Vero cells (C) were pretreated with mouse IFN-β (A) or human IFN-β (B and C) at different concentrations as indicated for 16 hr. Cells were then infected with Candid#1 virus at an MOI of 3. Cell lysates were prepared at 1 and 2 days p.i. from MEF cells and A549 cells, or at 2 days p.i. from Vero cells. The viral NP protein was detected with a monoclonal mouse anti-JUNV NP antibody (AG12, BEI) by Western Blotting assay. Equal loading of samples was confirmed by immunoblotting of the same membranes with an antibody to β-actin protein (Santa Cruz). Relative NP protein level in A549 cell samples is shown (B) after densitometry measurement and normalization to the actin protein level.

Next, we studied the effect of IFN on viral replication to understand the mechanism of IFN-induced antivirus activity. MEF cells were pretreated with murine IFN-β at 1, 5, 10 and 50 U/ml followed by infection with Candid#1 JUNV at an MOI of 3. Our data clearly showed that treatment with 1 U/ml of IFN-β almost abolished viral NP protein expression at days 1 and 2 p.i. (Fig 3A), consistent with the virus titration results (Fig 1A). NP protein is one of the early viral gene products expressed during virus infection [1]. Our result suggested that IFN probably targeted early steps of virus infection, such as virus entry, disassembly or early stages of viral RNA replication/transcription in mouse cells. In A549 cells, NP protein expression was inhibited by 90% in the presence of 500 U/ml human IFN-β (Fig 3B), which was consistent with the virus titration data (Fig 1C). In Vero cells, the synthesis of NP protein was moderately suppressed (Fig 3C) after 1000 U/ml human IFN-β treatment, in agreement with the relative resistance of JUNV to IFN treatment in this cell line (Fig 1B).

## Discussion

Overall our studies revealed the high sensitivity of JUNV to IFN as well as the attenuated growth of Candid#1 virus in primary murine cells. Treatment with 1 U/ml of IFN-β resulted in a drastic decrease in JUNV titers (over 3-log reduction for Candid#1 and 2.7-log for Romero), similar to the inhibitory effect of IFN on the IFN-sensitive VSV (Fig 1A). Expression of NP protein was abolished in the presence of exogenous 1 U/ml IFN-β for Candid#1 JUNV in murine cells at MOI 3. This result also suggested that the early stage of the virus replication was efficiently suppressed by IFN in murine cells. Known examples of IFN-induced gene products that could inhibit virus replication at early life cycle include: 1) MxA, which inhibits viral nucleocapsid shuttling and primary transcription of influenza virus, HCV and VSV [22]; 2) IFITMs, which inhibit the entry of influenza A virus, SARS coronavirus and West Nile virus [23] [24] and 3) IFIT1/2/3/5 complex, which blocks the replication of certain strains of WNV [25] and Venezuelan equine encephalitis virus [26] by targeting viral RNA either lacking 2′*O*-methylated cap structure or containing tri-phosphate group at the 5′-end. Future studies are required to identify which steps of virus replication are blocked by IFN treatment, as such study will help us better understand the JUNV-host interaction and will also facilitate the design of antiviral strategies.

Interestingly for some IFN-sensitive LCMV strains (the WE and Armstrong strains), treatment with a higher dose of murine IFN-α/β (100 u/ml) leads to about 2-log reduction in virus titer in murine cells [19]. The capacity of LCMV strains to establish persistent infection in adult immunocompetent mice has also been correlated with their relative resistance to IFN-α/β and IFN-γ [19]. For Lassa virus, treatment with high concentration of human IFN-α (1000 U/ml) leads to approximately 2-log reduction in virus titer in human cells [27]. Because of the difference in experimental conditions, it is difficult to directly compare the IFN sensitivity of JUNV in MEF cells with those results in aforementioned studies for LCMV and LASV. However, JUNV is apparently highly sensitive to IFN in murine cells, which could at least partially explain the mouse resistance to JUNV as discussed below. Since both human pathogenic and vaccine strains of JUNV are highly susceptible to IFN in murine cells as identified in this study, no correlation could be established between the pathogenicity of JUNV in humans and the IFN sensitivity in murine cells.

While relatively resistant to IFN in Vero cells, JUNVs were more susceptible to IFN in A549 cells. Based on the two-step positive-feedback loop model for IFN production [28], IFN-β and IFN-α4 are produced upon virus infection at the first step and secreted to induce IRF7 expression. At the next stage, activated IRF7 further stimulates the expression of other IFNs and allows the cells to mount a full scale antiviral response. Accordingly, it is possible that exogenous IFN-β could induce the synthesis of various subtypes of endogenous IFN in IFN-production competent A549 cells, resulting in a robust and sustained antiviral response. In Vero cells, due to its defect in IFN-β and IFN-α genes [29], the IFN-mediated antiviral response could be less potent as in A549 cells.

The high sensitivity of JUNVs to IFN in murine cells might explain in part the requirement of intact IFN pathway for adult mice to be resistant to JUNV. Macrophages are known as one of the initial targets of JUNV infection *in vivo* [8], [30], [31]. Mouse macrophages are found to produce IFN and other cytokines in response to infection by Candid#1 virus and presumably by pathogenic JUNV as a result of host recognition of viral glycoprotein protein in a TLR-2-dependent manner [32]. Considering the high IFN sensitivity of JUNV in murine cells as identified herein, it is possible that productive viral infection might be suppressed directly by IFN-induced antiviral gene products in macrophages or other cells. Moreover, induced IFN and cytokines could also activate different immune cells to promote JUNV clearance in vivo. These host barriers could be detrimental to JUNV dissemination in mice at the initial stage of viral infection, which might eventually render adult mice resistant to JUNV. In the absence of functional IFN pathway, the Romero JUNV is able to establish successful virus infection and become pathogenic in mice [17].

The role of IFN response in JUNV pathogenesis is still not well understood. High levels of IFN-α have been detected in serum samples from Argentine hemorrhagic fever (AHF) patients and have been associated with severe and lethal disease outcomes [9]. IFN has been linked to some of the clinical symptoms including thrombocytopenia [33]. In natural rodent host, the role of IFN in JUNV pathogenesis and persistent infection remains unclear, largely due to lack of laboratory inbred animals [13].

Although the mouse is not the natural host for JUNV, our results with murine cells provide some insights into the basis for Candid#1 virus attenuation. Candid#1-specific mutations leading to virus attenuation in humans and guinea pigs are not established. Its ancestor XJ#44 strain, which was established by 44 passages of the human pathogenic XJ strain in mouse brain [34], is attenuated for humans and guinea pigs but still virulent for suckling mice when introduced intracranially. The vaccine Candid#1 strain was established after additional passages of the XJ#44 strain in FRhL-2 cells. Attenuation of Candid#1 has been well characterized with a 14-day-old mouse model in a recent genetic study [35], in which the viral GPC glycoprotein has been found as the main determinant of JUNV virulence in mice. Among a total of six amino acid changes in Candid#1 virus sequence as compared with the XJ44 strain [35], [36], a single F427I substitution in the transmembrane region of GPC is sufficient for JUNV attenuation in suckling mice [35]. However, the mechanism of attenuation or the possible effect of accumulated mutations on virus replication in murine systems has not been established. We demonstrated the impaired growth of Candid#1 virus in primary murine cells, which was more evident at an MOI of 0.001. This defect might relate to the increased dependency of Candid#1 glycoprotein on human transferrin receptor for virus entry [36], the compromised efficiency of Candid#1 NP and L proteins in supporting viral RNA transcription/replication [37] or other mechanisms remained to be identified in future studies. A systemic characterization of the effect of Candid#1-specific mutations on virus replication in murine systems is warranted by utilizing the JUNV reverse genetic systems. The impaired virus growth in murine cells for Candid#1 strain is biologically relevant to its attenuation, as it could at least in part explain the inability of Candid#1 virus to cause disseminated infection in mice lacking functional IFN system (our unpublished observation).

## References

[1] Buchmeier MJ, de la Torre J-C, Peters CJ (2007) 51. Arenaviridae: The Viruses and Their Replication. In: Knipe DM, editor.Fields' Virology.Philadelphia: Lippincott, Williams and Wilkins. pp. 1635–1668.

[2] Stenglein MD, Sanders C, Kistler AL, Ruby JG, Franco JY, et al (2012) Identification, characterization, and in vitro culture of highly divergent arenaviruses from boa constrictors and annulated tree boas: candidate etiological agents for snake inclusion body disease. MBio 3: e00180–00112. 22893382 10.1128/mBio.00180-12 PMC3419519

[3] Bodewes R, Kik MJ, Raj VS, Schapendonk CM, Haagmans BL, et al (2013) Detection of novel divergent arenaviruses in boid snakes with inclusion body disease in The Netherlands. J Gen Virol 94: 1206–1210. 23468423 10.1099/vir.0.051995-0

[4] Hetzel U, Sironen T, Laurinmaki P, Liljeroos L, Patjas A, et al (2013) Isolation, identification, and characterization of novel arenaviruses, the etiological agents of boid inclusion body disease. J Virol 87: 10918–10935. 23926354 10.1128/JVI.01123-13 PMC3807292

[5] Peters CJ (2002) Human infection with arenaviruses in the Americas. Curr Top Microbiol Immunol 262: 65–74. 11987808 10.1007/978-3-642-56029-3_3

[6] Koma T, Huang C, Kolokoltsova OA, Brasier AR, Paessler S (2013) Innate immune response to arenaviral infection: a focus on the highly pathogenic new world hemorrhagic arenaviruses. J Mol Biol 425: 4893–4903. 24075870 10.1016/j.jmb.2013.09.028 PMC3864108

[7] McCormick JB, Fisher-Hoch SP (2002) Lassa fever. Curr Top Microbiol Immunol 262: 75–109. 11987809 10.1007/978-3-642-56029-3_4

[8] Geisbert TW, Jahrling PB (2004) Exotic emerging viral diseases: progress and challenges. Nat Med 10: S110–121. 15577929 10.1038/nm1142

[9] Levis SC, Saavedra MC, Ceccoli C, Feuillade MR, Enria DA, et al (1985) Correlation between endogenous interferon and the clinical evolution of patients with Argentine hemorrhagic fever. J Interferon Res 5: 383–389. 4056485 10.1089/jir.1985.5.383

[10] Maiztegui JI (1975) Clinical and epidemiological patterns of Argentine haemorrhagic fever. Bull World Health Organ 52: 567–575. 1085212 PMC2366633

[11] Enria DA, Briggiler AM, Sanchez Z (2008) Treatment of Argentine hemorrhagic fever. Antiviral Res 78: 132–139. 18054395 10.1016/j.antiviral.2007.10.010 PMC7144853

[12] Grant A, Seregin A, Huang C, Kolokoltsova O, Brasier A, et al (2012) Junin Virus Pathogenesis and Virus Replication. Viruses-Basel 4: 2317–2339. 10.3390/v4102317 PMC3497054 23202466

[13] Gomez RM, Jaquenod de Giusti C, Sanchez Vallduvi MM, Frik J, Ferrer MF, et al (2011) Junin virus. A XXI century update. Microbes Infect 13: 303–311. 21238601 10.1016/j.micinf.2010.12.006

[14] Weissenbacher MC, Laguens RP, Coto CE (1987) Argentine hemorrhagic fever. Curr Top Microbiol Immunol 134: 79–116. 3034513 10.1007/978-3-642-71726-0_4

[15] Ambrosio A, Saavedra M, Mariani M, Gamboa G, Maiza A (2011) Argentine hemorrhagic fever vaccines. Hum Vaccin 7: 694–700. 21451263 10.4161/hv.7.6.15198

[16] McKee KT Jr, Oro JG, Kuehne AI, Spisso JA, Mahlandt BG (1992) Candid No. 1 Argentine hemorrhagic fever vaccine protects against lethal Junin virus challenge in rhesus macaques. Intervirology 34: 154–163. 1338783 10.1159/000150276

[17] Kolokoltsova OA, Yun NE, Poussard AL, Smith JK, Smith JN, et al (2010) Mice lacking alpha/beta and gamma interferon receptors are susceptible to junin virus infection. J Virol 84: 13063–13067. 20926559 10.1128/JVI.01389-10 PMC3004311

[18] Yun NE, Linde NS, Dziuba N, Zacks MA, Smith JN, et al (2008) Pathogenesis of XJ and Romero strains of Junin virus in two strains of guinea pigs. Am J Trop Med Hyg 79: 275–282. 18689636 PMC2700623

[19] Moskophidis D, Battegay M, Bruendler MA, Laine E, Gresser I, et al (1994) Resistance of Lymphocytic Choriomeningitis Virus to Alpha/Beta Interferon and to Gamma-Interferon. Journal of Virology 68: 1951–1955. 8107255 10.1128/jvi.68.3.1951-1955.1994 PMC236657

[20] Huang C, Kolokoltsova OA, Yun NE, Seregin AV, Poussard AL, et al (2012) Junin virus infection activates the type I interferon pathway in a RIG-I-dependent manner. PLoS Negl Trop Dis 6: e1659. 22629479 10.1371/journal.pntd.0001659 PMC3358329

[21] Lazear HM, Lancaster A, Wilkins C, Suthar MS, Huang A, et al (2013) IRF-3, IRF-5, and IRF-7 Coordinately Regulate the Type I IFN Response in Myeloid Dendritic Cells Downstream of MAVS Signaling. Plos Pathog 9: e1003118. 23300459 10.1371/journal.ppat.1003118 PMC3536698

[22] Haller O, Kochs G (2011) Human MxA protein: an interferon-induced dynamin-like GTPase with broad antiviral activity. J Interferon Cytokine Res 31: 79–87. 21166595 10.1089/jir.2010.0076

[23] Perreira JM, Chin CR, Feeley EM, Brass AL (2013) IFITMs restrict the replication of multiple pathogenic viruses. J Mol Biol 425: 4937–4955. 24076421 10.1016/j.jmb.2013.09.024 PMC4121887

[24] Diamond MS, Farzan M (2013) The broad-spectrum antiviral functions of IFIT and IFITM proteins. Nat Rev Immunol 13: 46–57. 23237964 10.1038/nri3344 PMC3773942

[25] Daffis S, Szretter KJ, Schriewer J, Li J, Youn S, et al (2010) 2′-O methylation of the viral mRNA cap evades host restriction by IFIT family members. Nature 468: 452–456. 21085181 10.1038/nature09489 PMC3058805

[26] Hyde JL, Gardner CL, Kimura T, White JP, Liu G, et al (2014) A Viral RNA Structural Element Alters Host Recognition of Nonself RNA. Science 343: 783–787. 24482115 10.1126/science.1248465 PMC4209899

[27] Asper M, Sternsdorf T, Hass M, Drosten C, Rhode A, et al (2004) Inhibition of Different Lassa Virus Strains by Alpha and Gamma Interferons and Comparison with a Less Pathogenic Arenavirus. Journal of Virology 78: 3162–3169. 14990737 10.1128/JVI.78.6.3162-3169.2004 PMC353741

[28] Honda K, Takaoka A, Taniguchi T (2006) Type I inteferon gene induction by the interferon regulatory factor family of transcription factors. Immunity 25: 349–360. 16979567 10.1016/j.immuni.2006.08.009

[29] Desmyter J, Melnick JL, Rawls WE (1968) Defectiveness of interferon production and of rubella virus interference in a line of African green monkey kidney cells (Vero). J Virol 2: 955–961. 4302013 10.1128/jvi.2.10.955-961.1968 PMC375423

[30] Ambrosio M, Vallejos A, Saavedra C, Maiztegui JI (1990) Junin virus replication in peripheral blood mononuclear cells of patients with Argentine haemorrhagic fever. Acta Virol 34: 58–63. 1975726

[31] Ambrosio AM, Enria DA, Maiztegui JI (1986) Junin virus isolation from lympho-mononuclear cells of patients with Argentine hemorrhagic fever. Intervirology 25: 97–102. 3013799 10.1159/000149662

[32] Cuevas CD, Lavanya M, Wang E, Ross SR (2011) Junin virus infects mouse cells and induces innate immune responses. J Virol 85: 11058–11068. 21880772 10.1128/JVI.05304-11 PMC3194972

[33] Pozner RG, Ure AE, Jaquenod de Giusti C, D'Atri LP, Italiano JE, et al (2010) Junin virus infection of human hematopoietic progenitors impairs in vitro proplatelet formation and platelet release via a bystander effect involving type I IFN signaling. PLoS Pathog 6: e1000847. 20419155 10.1371/journal.ppat.1000847 PMC2855331

[34] Albarino CG, Ghiringhelli PD, Posik DM, Lozano ME, Ambrosio AM, et al (1997) Molecular characterization of attenuated Junin virus strains. J Gen Virol 78 (Pt 7): 1605–1610. 10.1099/0022-1317-78-7-1605 9225036

[35] Albarino CG, Bird BH, Chakrabarti AK, Dodd KA, Flint M, et al (2011) The major determinant of attenuation in mice of the Candid1 vaccine for Argentine hemorrhagic fever is located in the G2 glycoprotein transmembrane domain. Journal of Virology 85: 10404–10408. 21795336 10.1128/JVI.00856-11 PMC3196416

[36] Droniou-Bonzom ME, Reignier T, Oldenburg JE, Cox AU, Exline CM, et al (2011) Substitutions in the glycoprotein (GP) of the Candid#1 vaccine strain of Junin virus increase dependence on human transferrin receptor 1 for entry and destabilize the metastable conformation of GP. Journal of Virology 85: 13457–13462. 21976641 10.1128/JVI.05616-11 PMC3233171

[37] Emonet SF, Seregin AV, Yun NE, Poussard AL, Walker AG, et al (2011) Rescue from cloned cDNAs and in vivo characterization of recombinant pathogenic Romero and live-attenuated Candid #1 strains of Junin virus, the causative agent of Argentine hemorrhagic fever disease. J Virol 85: 1473–1483. 21123388 10.1128/JVI.02102-10 PMC3028888

